# Association between systemic immunity-inflammation index and psoriasis among outpatient US adults

**DOI:** 10.3389/fimmu.2024.1368727

**Published:** 2024-06-04

**Authors:** Qike Ding, Xiaoting Li, Lihong Lin, Xiaoping Xie, Wenjuan Jing, Xinyu Chen, Jiadong Chen, Tao Lu

**Affiliations:** Department of Dermatology, The First Affiliated Hospital of Shantou University Medical College, Shantou, Guangdong, China

**Keywords:** psoriasis, systemic immune-inflammatory index (SII), cross-sectional study, National Health and Nutrition Examination Survey (NHANES), outpatient US adults

## Abstract

**Background:**

Psoriasis is a chronic dermatological condition characterized by a complex pathogenesis that impacts approximately 3% of adults in the United States and brings enormous social burdens. For many diseases, the systemic immune-inflammatory index (SII), defined as neutrophils × platelets/lymphocytes, has been recognized as a prognostic indicator. Therefore, we conducted a cross-sectional study to assess the association between SII and psoriasis among outpatient US adults.

**Methods:**

In this cross-sectional study, we used data on the US adults 20 to 59 years of age from the National Health and Nutrition Examination Survey (NHANES) spanning 2003–2006 and 2009–2014. Sample-weighted logistic regression and stratified analysis of subgroups were used.

**Results:**

Among the 16,831 adults, there were 8,801 women and 8,030 men, with a psoriasis prevalence rate of 3.0%. A fully adjusted model revealed a positive association between a SII higher than 479.15 × 10^9^/L and a high risk of psoriasis. According to subgroup analysis and interaction testing (p for interaction > 0.05), age, sex, alcohol drinking status, marital status, and body mass index (BMI) were not significantly correlated with this positive association.

**Conclusion:**

Our findings suggested that SII higher than 479.15 × 10^9^/L was positively associated with a high risk of psoriasis among outpatient US adults. To the best of our knowledge, this is the first cross-sectional study using NHANES data focused on the risk of higher SII on psoriasis among outpatient US adults. The outcomes of this cross-sectional serve to supplement previous research, indicating a need for larger-scale prospective cohorts for further validation.

## 1 Introduction

Psoriasis is a chronic dermatological condition characterized by a complex pathogenesis and impacts approximately 3% of adults 20 years or older in the United States (1, 2). Research findings suggest that the prevalence of psoriasis among adults in the United States has remained relatively stable since 2003, showing no significant differences (2). Furthermore, psoriasis imposes considerable financial and social burdens, accounting for the largest global burden of disability due to skin diseases (3).

In the systemic inflammatory system of the human body, immune cells play a critical role in various diseases. Many studies have raised that integrated peripheral lymphocyte, neutrophil, and platelet counts may be more effective in predicting the inflammatory state. Importantly, SII, which possesses considerable clinical application, was calculated by counting three types of circulating immune cells: lymphocytes, platelets, and neutrophils (4, 5). Moreover, SII was initially identified as a prognosis of kidney stones, hepatic steatosis, and cancer investigations (6). Psoriasis is a chronic skin disorder with systemic involvement and inflammatory etiopathogenesis. The treatment of psoriatic skin lesions has seen notable effectiveness with interleukin-17 (IL-17), IL-23, and tumor necrosis factor-alpha (TNF-α) inhibitors (7). However, the impact of SII on psoriasis in the outpatient US population is not completely clarified, and its prognostic ability for psoriasis remains largely unknown. We hypothesized that SII could predict the risk of psoriasis, and we aimed to investigate the association between SII and psoriasis.

## 2 Methods

### 2.1 Data sources

Information on psoriasis was only provided in the National Health and Nutrition Examination Survey (NHANES) 2003–2006 cycles for individuals aged 20–59 and in the 2009–2014 cycles for individuals aged 16–80. In this cross-sectional study, deidentified data for participants aged 20–59 were extracted from the NHANES 2003–2006 and 2009–2014 cycles. The study was deemed exempt by the Shantou University Medical College institutional review board as it used publicly available deidentified data, and informed consent was waived. This study followed the Strengthening the Reporting of Observational Studies in Epidemiology (STROBE) reporting guideline. Data were analyzed from July to November 2023.

### 2.2 Study design and population

**Figure 1:**
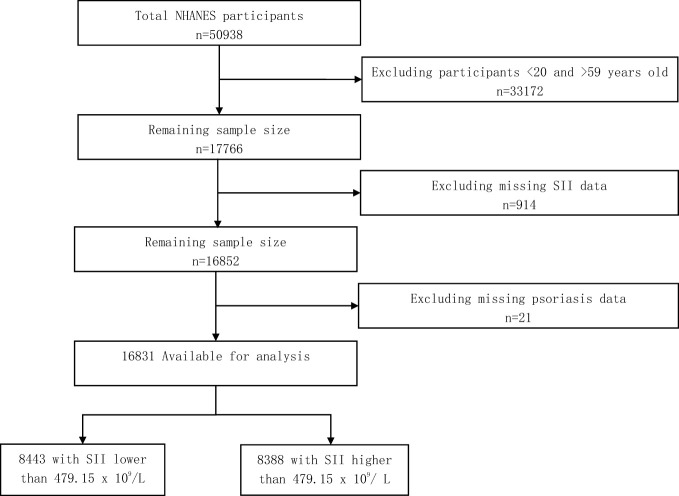
Flow diagram of the screening and enrollment of study participants.

From the NHANES 2003–2006 and 2009–2014 cycles, a total of 50,938 participants were enrolled. First, we selected 17,766 participants aged 20–59. Subsequently, we excluded 914 participants with missing SII data and 21 participants with missing psoriasis data. Finally, a total of 16,831 participants were involved. Of the 16,831 participants, there were 8,443 participants with SII lower than 479.15 × 10^9^/L and 8,388 participants with SII higher than 479.15 × 10^9^/L. The flowchart of participant enrollment is presented in **Figure 1**.

### 2.3 Assessment of SII and psoriasis

Psoriasis was defined as an affirmative response to the question, “Have you ever been told by a healthcare provider that you had psoriasis?” The SII was calculated using the peripheral neutrophil, lymphocyte, and platelet counts, defined as (platelet count × neutrophil count)/lymphocyte count (8). For all the analyses, we set the cutoff value at 479.15 × 10^9^/L (9).

### 2.4 Covariates

The following covariates were included in this cross-sectional study: sociodemographic variables (age, sex, race and ethnicity, family income, educational level, and marital status), NHANES cycles, body mass index (BMI), alcohol drinking status, and smoking status (10–12). First, races and ethnicities were categorized into the following four groups: Mexican American, non-Hispanic White, non-Hispanic Black, and Other. Based on the family poverty income ratio, family income was categorized into the following three levels: low income (≤1.3), medium income (>1.3 to 3.5), and high income (>3.5). Educational level was categorized into the following three levels: high school or less, some college, and college graduate or higher. Marital status was categorized into the following two groups: married and not married. Smoking status was categorized into the following three groups: never smoked (or smoked <100 cigarettes), former smoker (smoked at least 100 cigarettes but has quit), and current smoker (still smoking) (13). Finally, alcohol drinking status was determined by the question, “In any 1 year, have you had at least 12 drinks of any type of alcoholic beverage?” Participants who answered “yes” were defined as alcohol drinkers (14).

### 2.5 Statistical analysis

In accordance with NHANES analytic guidelines, our analyses considered the complex sampling design and sampling weights (15). We calculated the sampling weight using the following formula: fasting subsample 10-year mobile examination center (MEC) weight = fasting subsample 2-year MEC weight/5 (14). We used quartiles to describe the continuous variables with non-normal distribution and performed the Wilcoxon rank-sum test for comparison. We described the categorical variables using unweighted frequency and weighted percentage and conducted a chi-square test for comparison.

We checked for multicollinearity using the variance inflation factor (VIF) method. If the VIF was 5 or higher, it meant there was multicollinearity present. Model 1 was adjusted for sociodemographic variables and NHANES cycles. Model 2 was adjusted for sociodemographic variables, NHANES cycles, BMI, alcohol drinking status, and smoking status. Additionally, we conducted interaction and subgroup analyses using logistic regression models, stratified by age group, sex, alcohol drinking status, marital status, and BMI group.

We used R version 4.3.1 for all the statistical analyses, and we considered a significance level of p < 0.05 to show that the results were statistically significant.

## 3 Results

This cross-sectional study involved 50,938 participants from 2003–2006 and 2009–2014. First, 33,172 participants <20 and >59 years old were excluded. Then, 914 adults were excluded for missing SII data. After excluding 21 adults with missing psoriasis data, 16,831 participants were finally enrolled. As shown in **Table 1**, the prevalence rate of psoriasis was 3.0% in this cross-sectional study. Of the 16,831 adults, there were 8,801 women and 8,030 men. Compared with SII lower than 479.15 × 10^9^/L, adults with SII higher than 479.15 × 10^9^/L were more likely to be non-Hispanic White, to be current smokers, and to have an educational level of high school or less.

**Table 1:** Characteristics of participants in the NHANES 2003–2006 and 2009–2014 cycles.

Characteristic	Participants^a^	SII (10^9^/L)		p-Value
	Total (N = 16,831)	<479.15 (N = 8,443)	≥479.15 (N = 8,388)	
Age (median [IQR])	40.00 [30.00, 49.00]	39.00 [29.00, 50.00]	40.00 [30.00, 49.00]	0.375
BMI (median [IQR])	27.41 [23.84, 32.10]	26.86 [23.48, 31.20]	28.10 [24.19, 33.04]	<0.001
Sex				<0.001
Male	8,030 (49.1)	4,515 (54.2)	3,515 (44.2)	
Female	8,801 (50.9)	3,928 (45.8)	4,873 (55.8)	
Marital status				0.013
Married	8,545 (54.7)	4,322 (56.1)	4,223 (53.4)	
Not married	8,286 (45.3)	4,121 (43.9)	4,165 (46.6)	
Educational level				0.012
High school or less	7,546 (38.1)	3,694 (37.2)	3,852 (39.1)	
Some college	5,309 (33.0)	2,663 (32.5)	2,646 (33.5)	
College graduate or higher	3,963 (28.9)	2,081 (30.4)	1,882 (27.4)	
Race and ethnicity^b^				<0.001
Mexican American	2,939 (9.7)	1,346 (9.4)	1,593 (9.9)	
Non-Hispanic White	7,220 (65.8)	3,183 (62.1)	4,037 (69.4)	
Non-Hispanic Black	3,610 (11.8)	2,267 (15.1)	1,343 (8.6)	
Other	3,062 (12.7)	1,647 (13.4)	1,415 (12.0)	
Family income^c^				0.577
Low	5,236 (22.8)	2,573 (22.4)	2,663 (23.1)	
Medium	5,435 (34.3)	2,706 (34.2)	2,729 (34.4)	
High	5,033 (42.9)	2,587 (43.4)	2,446 (42.5)	
Alcohol drinker^d^				0.563
No	5,509 (20.6)	2,787 (20.4)	2,722 (20.8)	
Yes	11,322 (79.4)	5,656 (79.6)	5,666 (79.2)	
Smoking status^e^				0.001
Never	9,627 (55.6)	4,979 (57.3)	4,648 (53.9)	
Former	2,902 (19.3)	1,421 (19.2)	1,481 (19.5)	
Current	4,295 (25.1)	2,039 (23.5)	2,256 (26.6)	
Psoriasis^f^				<0.001
Yes	438 (3.0)	174 (2.3)	264 (3.7)	
No	16,393 (97.0)	8,269 (97.7)	8,124 (96.3)	

NHANES, National Health and Nutrition Examination Survey; BMI, body mass index (calculated as kilograms divided by meters squared); SII, systemic immune-inflammatory index; IQR, interquartile range.

a Data are presented as unweighted number (weighted percentage) unless otherwise indicated.

b Race and ethnicity were self-reported.

c Categorized into the following three levels based on the family poverty income ratio: low income (≤1.3), medium income (>1.3 to 3.5), and high income (>3.5).

d Determined by answering the following question: “In any 1 year, have you had at least 12 drinks of any type of alcoholic beverage?”

e Smoking status was categorized into the following three groups: never smoked (or smoked <100 cigarettes), former smoker (smoked at least 100 cigarettes but has quit), and current smoker (still smoking).

f Psoriasis was defined as an affirmative response to the question, “Have you ever been told by a healthcare provider that you had psoriasis?”

The results of the sample-weighted logistic regression analyses are displayed in **Table 2**. A positive association between a SII higher than 479.15 × 10^9^/L and a high risk of psoriasis was revealed in model 2 (OR, 1.55; 95% CI, 1.20–2.01, p = 0.001).

**Table 2:** Association of SII with psoriasis among participants in the NHANES 2003–2006 and 2009–2014 cycles.

	Unweighted participants/total participants, no.		OR (95% CI)	p-Value
	SII ≥ 479.15	SII < 479.15		
**Crude model**	264/8,388	174/8,443	1.63 (1.29–2.07)	<0.001
**Model 1** **^a^**	247/7,830	167/7,859	1.58 (1.22–2.04)	<0.001
**Model 2** **^b^**	225/7,067	149/7,030	1.55 (1.20–2.01)	0.001

NHANES, National Health and Nutrition Examination Survey; OR, odds ratio; SII, systemic immune-inflammatory index; BMI, body mass index.

a Adjusted for sociodemographic variables (age, sex, race and ethnicity, family income, educational level, and marital status) and NHANES cycles.

b Adjusted for sociodemographic variables, NHANES cycles, BMI, alcohol drinking status, and smoking status.

**Figure 2:**
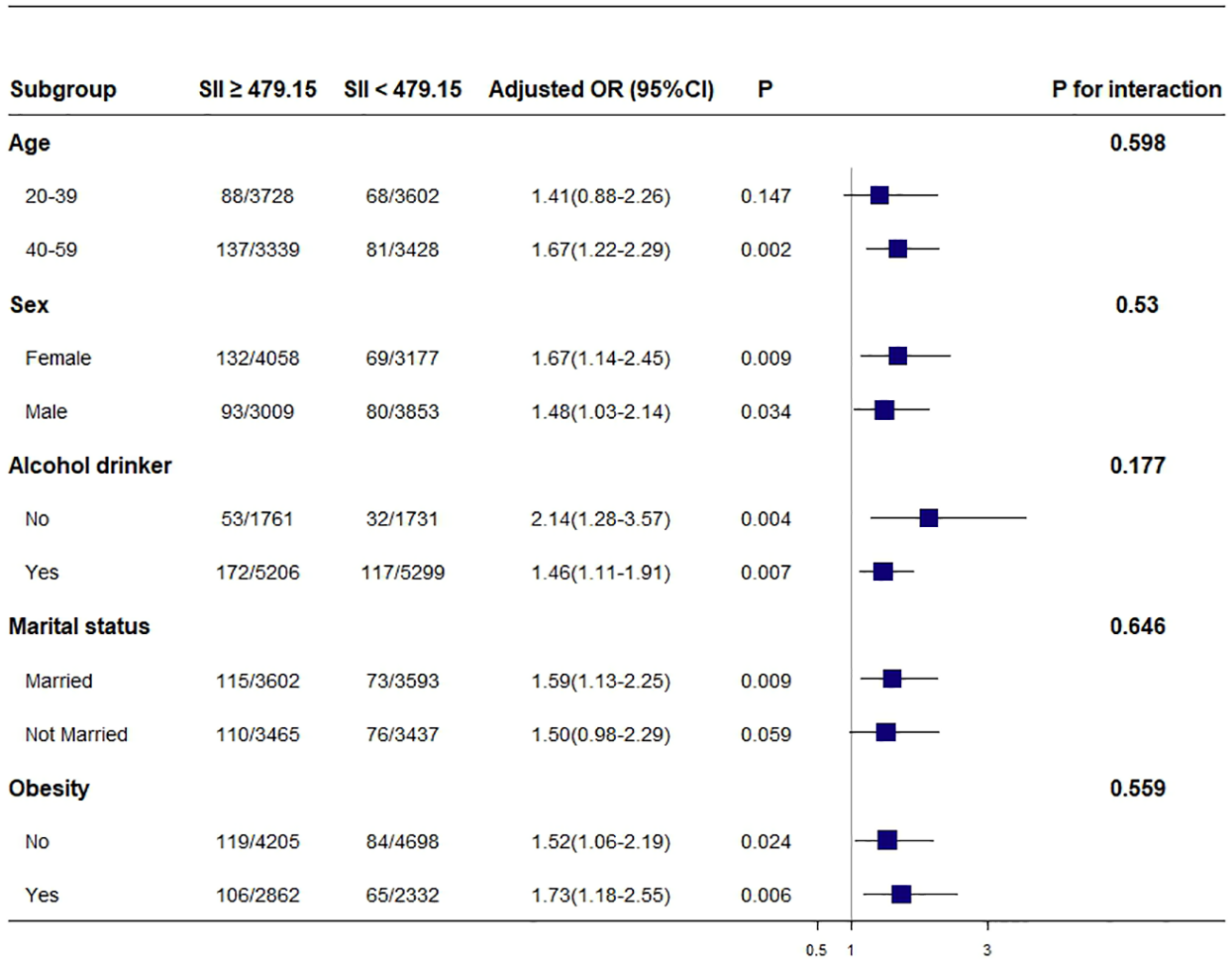
Association between SII and psoriasis. Each stratification was adjusted for age, sex, race and ethnicity, family income, educational level, marital status, NHANES cycles, BMI, alcohol drinking status, and smoking status except the stratification factor itself. Squares indicate odds ratios (ORs), with horizontal lines indicating 95% CIs. Obesity was defined as BMI ≥ 30. SII, systemic immune-inflammatory index; NHANES, National Health and Nutrition Examination Survey; BMI, body mass index.

We found that SII was not positively associated with psoriasis among participants aged 20–39 (OR, 1.41; 95% CI, 0.88–2.26, p = 0.147) and those not married (OR, 1.50; 95% CI, 0.98–2.29, p = 0.059). In addition, we found that SII was positively associated with psoriasis in the sex, alcohol drinking status, and obesity subgroups. The results of the subgroup analyses are displayed in **Figure 2**.

## 4 Discussion

Many studies have found the significant contribution of chronic inflammation to the progression of psoriasis (16, 17). The immune system’s role as a prognostic determinant in the pathogenesis of many diseases has been recognized over a long period of time. SII was determined by tallying up the numbers of three different types of immune cells: lymphocytes, platelets, and neutrophils. The SII level, which reflects inflammatory responses, shows potential as a valuable diagnostic biomarker for evaluating overall inflammatory activity. A retrospective study suggests SII could function as a prognostic indicator for adults with psoriasis (18). However, it is still not clear if there is a link between SII levels and psoriasis among outpatient US adults. Our previous study, published in *JAMA Dermatology*, indicated that in outpatient US adults, psoriasis was positively associated with non-alcoholic fatty liver disease (NAFLD) (14). A recent study has shown a positive association between SII and hyperlipidemia among outpatient US adults (9). Hyperlipidemia is recognized as a risk factor for NAFLD. Therefore, it raises the question of whether there is a positive association between SII and psoriasis. In this cross-sectional study, our findings suggest that participants with SII higher than 479.15 × 10^9^/L had a higher risk of psoriasis after fully adjusting. Furthermore, SII characterized by a non-intrusive methodology, cost-effectiveness, and easy accessibility is a widely available method. Therefore, SII can be identified as a biomarker for psoriasis in the outpatient US adult population.

This is the first cross-sectional study focused on the risk of higher SII on psoriasis in the outpatient US population. Due to our consideration of the NHANES design for obtaining nationally representative estimates in the United States, our findings should be applicable to the outpatient population of US adults.

Our subgroup analysis showed that participants with SII higher than 479.15 × 10^9^/L and obesity had a higher risk of psoriasis. Due to the restricted sample size of participants in this cross-sectional study, it is advisable to interpret these results with caution, and more well-designed prospective studies are warranted.

## 5 Limitations

As a cross-sectional study, the reliability of drawing causal conclusions is not robust, and we cannot completely rule out the potential impact of residual confounding from unmeasured variables. While NHANES is a nationally representative survey, the generalizability of our conclusions to other countries is uncertain, and further research is needed to explore the precise associations among them.

## 6 Conclusions

Our findings suggest a positive association between a SII higher than 479.15 × 10^9^/L and a high risk of psoriasis among outpatient US adults. SII characterized by a non-intrusive methodology, cost-effectiveness, and easy accessibility is a widely available method. Therefore, SII can be identified as a biomarker for psoriasis in the outpatient US adult population.

## Data availability statement

The original contributions presented in the study are included in the article/supplementary material. Further inquiries can be directed to the corresponding author.

## Ethics statement

The studies involving humans were approved by National Center for Health Statistics (NCHS). The studies were conducted in accordance with the local legislation and institutional requirements. The participants provided their written informed consent to participate in this study.

## Author contributions

QD: Writing – original draft, Writing – review & editing. XL: Writing – review & editing, Writing – original draft. LL: Writing – review & editing, Writing – original draft. XX: Writing – original draft, Writing – review & editing. WJ: Writing – review & editing, Writing – original draft. XC: Writing – review & editing, Writing – original draft. JC: Writing – review & editing, Writing – original draft. TL: Writing – review & editing, Writing – original draft.
